# Surgical Management of Recurrence of Primary Intrahepatic Bile Duct Stones

**DOI:** 10.1155/2023/5158580

**Published:** 2023-01-23

**Authors:** HongTian Xia, HangYu Zhang, XianLei Xin, Bin Liang, Tao Yang, Yang Liu, Jing Wang, XiangFei Meng

**Affiliations:** Faculty of Hepato-Pancreato-Biliary Surgery, Chinese PLA General Hospital, Institute of Hepatobiliary Surgery of Chinese PLA, Beijing 100853, China

## Abstract

**Background:**

The surgical treatment of primary intrahepatic bile duct stones is associated with high rates of postoperative complications, stone recurrence, and reoperation. This study aimed to report an 11-year experience in the management of postoperative recurrence of intrahepatic bile duct stones, analyze the causes of the reoperation, and establish appropriate surgical procedures.

**Materials and Methods:**

The records of 148 patients with postoperative recurrence of primary intrahepatic bile duct stones treated from January 2005 to December 2015 were retrospectively reviewed. Prior surgical treatment and postoperative data were analyzed to investigate possible causes of recurrence and reoperation.

**Results:**

All patients with a prior cholangiojejunostomy (*n* = 61) developed biliary stenosis (100%). Of the 86 patients without cholangiojejunostomy, 71 (82.56%) had abnormalities in the structure and function of the lower end of the common bile duct, and 86 had hilar and intrahepatic bile duct stenosis. Of all 148 patients, 136 (91.89%) had positive intraoperative bile cultures. Patients were treated with a modified surgical procedure, and the combined excellent and good rate of long-term outcomes reached 85.48% (106/124). The stone recurrence rate of the 124 patients decreased from 100% (124/124) of the prior operation to 5.65% (7/124) during the reoperation.

**Conclusions:**

The pathogenesis of primary intrahepatic bile duct stones is associated with biliary infection and intrahepatic bile duct cholestasis. According to the etiology and pathogenic mechanism, surgical procedures that improve long-term outcomes and reduce postoperative recurrence include bile duct exploration with stone extraction, partial hepatectomy, hilar ductoplasty, and Roux-en-Y hepaticojejunostomy.

## 1. Introduction

Primary intrahepatic bile duct stones are defined as intrahepatic calculi that develop de novo within the intrahepatic ducts [1]. Primary intrahepatic bile duct stones are rare in Western countries [2], but are prevalent in the Asian population, especially in China [3]. The progression of this disease may lead to recurrent episodes of cholangitis that can progress to liver cirrhosis and even cholangiocarcinoma [4]. At present, the pathogenic mechanism of primary bile duct stones has not been fully understood, but it is believed to be related to bile tract infection and cholestasis [5–7]. Current treatments for primary intrahepatic bile duct stones include the removal of all biliary stones, the establishment of proper bile duct flow, and surgical resection of the affected liver parenchyma [8]. Nevertheless, because the etiology and pathogenesis are not fully understood, the outcomes of surgical treatment are still far from satisfactory, and surgery is associated with relatively high rates of postoperative stone recurrence, reoperation, and complications [9–11].

In the authors' center, a large number of reoperations have been performed for postoperative recurrence of primary intrahepatic bile duct stones. It was found that the main reason for postoperative recurrence and reoperation was that the surgeon adopted inappropriate surgical methods at the prior operation due to an insufficient understanding of the etiology and pathogenesis of the disease. Based on the experience accumulated over the years, a set of surgical approaches for recurrent intrahepatic bile duct stones has gradually matured at our center. The purpose of this study is to report an 11-year experience in the management of the postoperative recurrence of intrahepatic bile duct stones at a high-volume Chinese center.

## 2. Methods

### 2.1. Patients

A total of 148 patients with postoperative recurrence of primary intrahepatic bile duct stones were treated at the authors' center from January 2005 to December 2015, and their records were retrospectively reviewed. Recurrent bile duct stones were defined as a history of stone clearance after surgical treatment and the reoccurrence of intrahepatic or extrahepatic bile duct stones by imaging findings. All patients reported in the current study were diagnosed with postoperative recurrence of primary intrahepatic bile duct stones and required reoperation. Patients with secondary bile duct stones, severe liver dysfunction, biliary cirrhosis, hypersplenism, intolerance to routine and standardized surgical treatment, and malignant transformation after the primary surgery were excluded.

### 2.2. Data Extraction

The medical history of each patient was examined, and data were extracted from the medical records, including the initial diagnosis and diagnostic methods, type and distribution of intrahepatic bile duct stones before the prior operation, prior surgical methods, the clinical manifestations after the prior operation, the total number of operations and the methods of each operation, and the postoperative curative effect.

Data extracted regarding treatment at the authors' center included clinical history, serological examination results, imaging results including abdominal ultrasound, computed tomography (CT), and magnetic resonance cholangiopancreatography (MRCP), and the type and distribution of intrahepatic bile duct stones.

### 2.3. Surgical Methods

Based on research on the etiology and pathogenesis of intrahepatic bile duct stones, the operating surgeon used 2 major types of surgical methods. Surgical Method A: laparotomy, intrahepatic bile duct exploration and stone removal, extrahepatic bile duct resection, partial hepatectomy, hilar ductoplasty, and Roux-en-Y hepaticojejunostomy; surgical Method B: the same procedures as Method A, but the partial hepatectomy was eliminated. The surgical method was chosen according to medical history, prior operation methods, the distribution of intrahepatic bile duct stones, the location and extent of stenosis of the bile duct, the extent and scope of pathological changes in the liver, and the preoperative examination findings.

### 2.4. Intraoperative Biliary Exploration

A choledochoscope was used to comprehensively explore the intrahepatic and extrahepatic biliary systems, focusing on the structure and function of the duodenal papilla at the lower end of the common bile duct, stenosis of the hilar bile duct, and the original biliary-intestinal anastomosis. The intraoperative findings provided a reference for establishing proper bile flow in the subsequent procedure.

### 2.5. Intraoperative Assessment of Duodenal Papilla Structure and Function

There is currently no standard method for assessing the structure and function of the duodenal papilla. In this study, the assessment included intraoperative observation of the morphology of the duodenal papilla and the choledochoscopic pass-through test [12]. Morphological observation focused on the diameter of the common bile duct, inflammation of the inner wall of the common bile duct, the opening and closing function of the duodenal papilla, rhythmic contraction of the sphincter of Oddi, the confluence of the bile ducts and pancreatic duct, the presence of a fistula between the common bile duct and duodenum, and other obvious structural and functional abnormalities.

For the choledochoscopic pass-through test, 2 choledochoscopes with different diameters (P60 choledochoscope, outer diameter = 4.9 mm, Olympus, Japan; CD30s ultra-thin choledochoscope, outer diameter = 2.7 mm, Olympus) were used to determine if the choledochoscope could pass through the duodenal papilla into the duodenum without any expansion of the papilla. The assessment criteria were as follows. Normal function: The CD30 choledochoscope can pass through the duodenal papilla, but the P60 choledochoscope cannot.

  Incomplete closure of the duodenal papilla: the P60 choledochoscope can pass through the duodenal papilla. Papillary stenosis: Both P60 and CD30 choledochoscopes cannot pass through the duodenal papilla.

### 2.6. Hilar Ductoplasty and Roux-en-Y Hepaticojejunostomy

After the stones were removed and the biliary system was thoroughly explored, the common bile duct was radically excised as much as possible to avoid complications after biliary-enteric anastomosis. When resecting the lower end of the common bile duct, attention was given to not leaving dead space to prevent postoperative infection, cholangitis, and pancreatitis.

The purpose of extrahepatic bile duct resection is to address structure and function abnormalities of the sphincter of Oddi at the lower end of the common bile duct and hilar bile duct stenosis. Therefore, after extrahepatic bile duct resection, hilar ductoplasty [12] was performed to ensure the opening of the biliary-enteric anastomosis was of sufficient diameter to prevent postoperative complications. This was followed by a Roux-en-Y hepaticojejunostomy [12].

### 2.7. Indications and Contraindications of Partial Hepatectomy

The indications for partial hepatectomy were as follows: (1) the intrahepatic bile duct stones were strictly distributed within a specific liver segment or lobe and thus could be eliminated by partial hepatectomy; (2) the structure and function of the affected liver were damaged and would not recover after stone removal; (3) the strictured segment of the intrahepatic bile duct is at a relatively high site, and proper bile duct flow cannot be established with hilar ductoplasty; (4) after partial hepatectomy, the remaining liver will be of sufficient volume for normal liver function; and (5) intrahepatic bile duct stones are diffusely distributed. While partial hepatectomy cannot completely remove intrahepatic lesions, it will remove hepatic hilar bile duct stenosis to ensure proper bile duct flow in the remaining intrahepatic bile ducts.

The contraindications of partial hepatectomy were as follows: (1) the volume of the affected liver is large, and there will be insufficient remaining liver; (2) biliary cirrhosis and decompensated liver function; and (3) intrahepatic bile duct stones are widely distributed, the stricture site of the intrahepatic bile duct is at a relatively high level, or narrow bile ducts are widely distributed, and partial hepatectomy will not establish proper bile duct flow.

## 3. Short-Term Efficacy Evaluation

Short-term efficacy was assessed during hospitalization and included the success rate of the operation, the rate of stone clearance, the surgical duration, the amount of intraoperative blood loss, the amount of blood transfusion, and the length of the postoperative hospital stay. Postoperative complications recorded during hospitalization included hemorrhage, bile leakage, pancreatic leakage, abdominal infection, and delayed incision healing. In addition, the grade of short-term postoperative complications and management methods were recorded, as well as whether the symptoms resolved after surgery.

### 3.1. Postoperative Follow-Up

All patients were followed up at the authors' center or a local hospital. Patients who were found to have abnormalities at a local hospital were referred to the authors' center. Patients were followed up every 3 months for the first year, every 6 months for years 2–5, and then yearly. Patients with symptoms were evaluated as needed. Follow-up included evaluation of postoperative biliary function and symptoms such as cholangitis, abdominal pain, fever, and jaundice. Abdominal ultrasound, CT, and MRI were performed to screen for postoperative biliary-enteric anastomotic stricture, recurrence of bile duct stones, and postoperative malignant transformation.

### 3.2. Long-Term Efficacy Assessment

Assessment of long-term biliary function was based on the evaluation standard of Lillemoe et al. [13]. In brief, the biliary function was scored as follows: “excellent” if biochemical indicators were normal and there were no clinical symptoms or anatomical abnormalities; “good” if there were no clinical manifestations of cholangitis, only a few small bile ducts exhibited abnormal structure, biochemical tests were within normal ranges, and the patient did not require medical intervention; “fair” if mild anatomical abnormalities of the bile ducts were present and clinical manifestations of cholangitis were observed (<3 times/year), and they could be relieved by conservative treatments such as antibiotics; “poor” if cholangitis occurred repeatedly, the anatomical structure of the bile ducts was abnormal, bile duct strictures and stones were present, and reoperation was required. Postoperative complications were graded according to the Clavien-Dindo classification [14].

## 4. Results

### 4.1. Patient Demographic and Clinical Characteristics

Of the 148 patients, there were 62 males and 86 females, with a mean age of 46.37 ± 11.80 years (range 25–70 years). Patient demographic and clinical characteristics are shown in Table 1. All 148 patients were diagnosed with the recurrence of primary intrahepatic bile duct stones according to medical history, imaging findings (ultrasound, CT, MRI), and intraoperative findings. The classifications of stones before the prior operation included 93 patients with limited distribution (62.84%), 37 patients with diffuse stones (25.00%), and 18 patients of unknown type (12.16%).

### 4.2. Intraoperative Findings and Complications after the Prior Operation

Intraoperative bile duct exploration showed that of the 61 patients who received cholangiojejunostomy at the prior operation (surgical methods II and IV), 45 had biliary-enteric anastomotic stenosis (73.8%, 45/61) and 17 had intrahepatic bile duct stenosis (27.9%, 17/61). Among them, 21 had both biliary-enteric anastomotic stenosis and intrahepatic bile duct stenosis (46.67%, 21/45). This result indicates that all patients who received cholangiojejunostomy developed biliary stenosis (100%), but the stenotic segments varied. Therefore, biliary stenosis is an important factor for the postoperative recurrence of intrahepatic bile duct stones.

Intraoperative bile duct exploration showed that of the 86 patients without Roux-en-Y cholangiojejunostomy at the prior operation (surgical methods I and III), 71 (82.56%, 71/86) had abnormalities in the structure and function of the lower end of the common bile duct, and 22 (30.99%, 22/71) had stenosis at the lower end of the common bile duct. Forty-nine patients (69.01%, 49/71) had incomplete closure of the lower end of the common bile duct, and 86 (100%, 86/86) had hilar and intrahepatic bile duct stenosis. This result indicates that biliary system stenosis and structural abnormalities are common in cases of recurrent intrahepatic bile duct stones without biliary-enteric anastomosis at the prior operation. This result also suggests that abnormal structure and function of the biliary system are crucial factors for the postoperative recurrence of intrahepatic bile duct stones. It also indicates that the formation of intrahepatic bile duct stones is closely related to structural abnormalities of the biliary system.

Of all patients, 136 (91.89%, 136/148) had positive intraoperative bile cultures. The common pathogens were Gram-negative bacilli and anaerobic bacteria, similar to intestinal bacteria.

### 4.3. Distribution of Recurrent Intrahepatic Bile Duct Stones

The distribution of recurrent intrahepatic bile duct stones was different from the distribution before the prior operation. Imaging studies and intraoperative exploration showed that 76 patients (51.35%, 76/148) had limited distribution and 72 (48.65%, 72/148) had diffuse stones. Some cases of limited distribution changed to diffuse stones after the prior operation. This result indicates that the distribution of recurrent intrahepatic bile duct stones tends to expand after the prior operation, making treatment more difficult.

### 4.4. Methods of Reoperation

The method of reoperation was determined mainly based on the following factors: (1) clinicopathological classification of intrahepatic bile duct stones; (2) methods of prior operation; (3) extent of the affected liver; (4) location and severity of bile duct stenosis; and (5) comprehensive factors such as the patient's general condition and liver function. Two different reoperations were performed:

surgical Method A (bile duct exploration, extrahepatic bile duct resection, partial hepatectomy, hilar ductoplasty, and Roux-en-Y hepaticojejunostomy) was performed in 79 patients (53.38%), and surgical Method B (bile duct exploration, extrahepatic bile duct resection, hilar ductoplasty, and Roux-en-Y hepaticojejunostomy) was performed in 69 patients (46.62%). The methods of reoperation in patients with different stone distributions are shown in Table 2.

### 4.5. Short-Term Postoperative Outcomes

The reoperation was successfully completed in all 148 patients. The mean operation was 208.58 ± 45.67 minutes (range, 150–360 minutes). Only 5 patients received an intraoperative blood transfusion, and the blood transfusion volume was 200–600 mL per patient. After reoperation, all patients had resolution of symptoms, recovered, and were discharged. The mean postoperative hospital stay was 11.46 ± 2.58 days (range, 8–21 days). Intraoperative choledochoscopy was used for the exploration and removal of stones, and the stone removal rate was 100%.

Postoperative complications occurred in 19 patients during hospitalization, and the complication rate was 12.84% (19/148, Table 3). There were 6 cases of simple bile leakage, 11 cases of bile leakage with abdominal infection, 11 cases of delayed wound healing, and 4 cases of abdominal bleeding (Table 3). Bile leakage was resolved by drainage, and abdominal cavity infection was resolved by antibiotic treatment and abdominal cavity irrigation and drainage. Delayed wound healing was treated with standard wound care, and postoperative abdominal hemorrhage was treated by transhepatic artery interventional embolization. According to the Clavien-Dindo Classification of Surgical Complications, there were 14 cases of grade II complications and 5 cases of grade III complications. There were no grade IV or above complications.

### 4.6. Long-Term Postoperative Outcomes

Of the 148 patients, 124 received long-termfollow-up of more than 36 months, and the follow-up rate was 83.78% (124/148). The mean follow-up time was 98.98 ± 40.86 months (range, 36–168 months).

During follow-up, 18 patients developed recurrent cholangitis (14.52%, 18/124). Among the 18 patients, 11 had occasional transient cholangitis with mild symptoms, which improved with symptomatic treatment and antibiotics. The other 7 patients had more severe and frequent symptoms.

During the follow-up period, 7 patients developed bile duct stenosis and bile duct stones but recovered well after another operation. During the follow-up period, 3 patients died due to unrelated diseases. One patient died of cholangiocarcinoma secondary to the malignancy of the original hilar biliary-enteric anastomosis, accompanied by bile duct stones, which might be related to recurrent reflux cholangitis.

According to the classification and evaluation of postoperative efficacy, 86 patients had excellent (69.35%, 86/124) outcomes, 20 had good (16.13%, 20/124) outcomes, 11 had moderate outcomes (8.87%, 11/124), and 7 had poor (5.65%, 7/124) outcomes (Table 4). The long-term combined excellent and good rate was 85.48% (106/124). The total recurrence rate of bile duct stones was 5.65% (7/124).

During follow-up, 18 patients developed recurrent cholangitis, of whom 7 developed biliary-enteric anastomosis stenosis. Six patients received another operation, and the reoperation rate was 4.84% (6/124).

## 5. Discussion

Currently, studies on the efficacy of surgical treatments for recurrent intrahepatic bile duct stones are limited. Jeng et al. [15] conducted a case-control study to investigate the therapeutic outcomes of percutaneous dilatation of strictures and transhepatic percutaneous cholangioscopic lithotomy for recurrent intrahepatic bile duct stones. Among the 18 enrolled patients, 7 patients received an expandable metallic Z stent (group A), and 11 patients received repeated placement of external and internal stents (group B). During a follow-up period of 28 to 60 months, postoperative complications (recurrent cholangitis) were common (43% and 73% in groups A and B, respectively). Group B had a significantly higher rate of stone recurrence than group A (64% vs 0%; *P* = 0.01). Tian et al. [16] analyzed the efficacy of laparoscopic procedures in the treatment of intrahepatic bile duct stones, in which 38 out of 90 patients had previous biliary surgery. During a follow-up period of 3–51 months (mean = 19 months) for the 38 patients with previous biliary surgery, the postoperative complications rate was 18.4% and the stone recurrence rate was 7.9%. The author concluded that the laparoscopic approach is safe and feasible for intrahepatic bile duct stones in patients with previous biliary operations. Pu et al. [17] investigated the therapeutic effect of laparoscopy versus laparotomy for recurrent intrahepatic bile duct stones. Fifty-three patients received open biliary exploration, and 41 patients underwent laparoscopic biliary exploration. During the 1–51 months follow-up period (median = 12 months), 18 patients (19.1%) developed repeated cholangitis, and 7 cases (7.4%) had recurrent stones. Compared to these studies, the follow-up duration of this study was markedly longer (mean = 98.98, range 36–168 months). As for postoperative complications, 18 cases (14.5%) had cholangitis, and 2 patients (1.6%) had bile duct stricture. Only 7 (5.65%) patients had recurrent stones. In addition, the combined long-term excellent and good rate reached 85.48%. These results suggest that the surgical methods described in this study can effectively improve long-term outcomes as well as prevent severe postoperative complications and recurrent stones.

In this study, it was found that the prior surgical treatment for primary intrahepatic bile duct stones mainly focused on eliminating lesions and stones, and the operating surgeon paid little attention to infection control and the establishment of proper bile duct flow. Furthermore, partial hepatectomy is often overused. Improper surgical methods lead to postoperative repeated cholangitis, the recurrence of bile duct stones, and possibly cholangiocarcinoma. It is believed that the pathogenesis of primary intrahepatic bile duct stones is closely related to biliary tract infection and cholestasis [5, 6], and biliary tract infections include bacterial and parasitic infections [18]. Supporting this notion, this study showed that approximately 92% of patients had positive intraoperative bile cultures. It was also observed that approximately 75% of patients with primary bile duct stones had abnormalities in the structure and function of the lower end of the bile duct (intraoperative bile duct exploration, unpublished data). In addition, the incidence of structural and functional abnormalities of the sphincter of Oddi is relatively high in China [19], which is consistent with the high incidence of primary bile duct stones in China [20]. A reasonable pathogenic mechanism is that the sphincter of Oddi dysfunction causes duodenal bacteria to enter the bile duct system, and then growth in the bile duct segment with cholestasis leads to biliary infection. Repeated infections cause bile duct stenosis, which in turn aggravates cholestasis.

Based on the etiology and pathogenesis of primary bile duct stones, there are 4 principles for the surgical treatment of primary intrahepatic bile duct stones: (1) elimination of the lesions; (2) infection control; (3) elimination of the obstruction; and (4) establishment of proper bile duct flow. Elimination of the lesions includes removing the stones as much as possible and excision of the abnormal liver and bile duct (partial hepatectomy). Since the abnormal structure and function of the duodenal papilla cannot be repaired, a cholangiojejunostomy should be performed to block the source of bacteria [21]. Cholangiojejunostomy not only blocks the entry of intestinal bacteria into the biliary tract but also treats cholestasis caused by the structural abnormality of the sphincter of Oddi. The proper bile duct flow is established by surgical treatment to restore the physiological functions of the biliary system [22].

The purpose of partial hepatectomy is to ensure the radical treatment of intrahepatic bile duct stones and to avoid unnecessary expansion of the scope of surgery [23]. If the bile duct stenosis is located in the primary or secondary bile duct close to the hilum, hilar ductoplasty can be performed to relieve the bile duct obstruction. Based on the pathological condition of the affected liver, it may be possible to retain the affected liver instead of a partial hepatectomy. If bile duct stenosis is located at the opening of a secondary or tertiary bile duct in the liver and hilar ductoplasty is not possible, partial hepatectomy should be performed. Another consideration is the extent of the affected liver. If the affected liver has obvious fibrosis, atrophy, and evidence of ischemia or cirrhosis and normal function will not be restored after stone removal, obstruction elimination, and the establishment of proper bile duct flow, a partial hepatectomy should be performed [23, 24]. On the other hand, in mild disease, partial hepatectomy is not required when the function can be restored after stone removal and the establishment of proper bile duct flow. For inoperable diffuse intrahepatic bile duct stones and severe hilar bile duct stenosis, partial hepatectomy should be performed to remove the remaining stones and properly establish bile duct flow.

Choledochojejunostomy can treat bacterial infections in the case of intrahepatic and extrahepatic bile duct stones. However, the duodenal papillary sphincter often loses its antireflux function after biliary-enteric anastomosis, leading to severe complications, such as biliary-enteric anastomosis stenosis and hilar bile duct stenosis. Postcholedochojejunostomy hilar bile duct stenosis can be attributed to the anatomical structures of the hilar bile duct and the hilar plate [25]. Postcholedochojejunostomy reflux of intestinal fluid causes local infection/inflammation [22] and scar formation, eventually leading to hilar bile duct stenosis [26]. The bile duct stenosis leads to intrahepatic bile duct cholestasis, which further aggravates the biliary infection [22].

To reduce postcholedochojejunostomy complications, the scope of resection was expanded to include the hilar bile duct. Because the intrahepatic and extrahepatic bile ducts have separate blood supplies, resection of the extrahepatic bile duct reduces ischemia of the bile duct tissue. In addition, hilar ductoplasty was used to expand the diameter of the biliary-enteric anastomosis and eliminate the impact of fibrous connective tissue of the hilar plate. This modified Roux-en-Y hepaticojejunostomy has been performed at the authors' center for more than 10 years and achieves satisfactory therapeutic effects. The incidence of long-term complications of posthepaticojejunostomy decreased from about 30% (between 1993 and 2006, the long-term complications rate was 32.91% [51/158], unpublished data) to around 5% (after the modified procedure began to be performed) [12].

Based on patient data and intraoperative biliary exploration findings, the causes of postoperative recurrence of intrahepatic bile duct stones can be divided into 4 types.

(1) The biliary infection was not controlled. Although biliary anastomosis cannot completely block bacterial entry into the bile duct, it can significantly reduce the reflux of intestinal fluid and reduce the risk of infection. Some patients only received bile duct exploration with stone extraction in the previous operation, and biliary bacterial infection was not treated, eventually leading to postoperative recurrence of stones. In some cases of localized intrahepatic bile duct stones, no stones were found in the common bile duct, and the surgeon only removed the stones and performed partial hepatectomy to excise the affected part of the liver. However, the problem of bacterial infections due to the abnormal structure and function of the duodenal papilla at the lower end of the common bile duct remained unresolved. These findings suggest that unresolved bacterial infections inevitably cause postoperative recurrence of stones. (2) The lesion was not completely eliminated. Stones and diseased liver tissue affected by the stones need to be removed. Some cases of postoperative recurrence were because the severely diseased liver tissue was not excised, resulting in a continuous increase in histogenic *β*-glucuronidase secreted by the diseased liver, eventually leading to stone recurrence. In some cases, the surgeon removed recoverable liver tissue, resulting in unnecessary damage and a more difficult postoperative recovery. (3) Unresolved bile duct stenosis. Bile duct stenosis is a necessary condition for the formation of primary intrahepatic bile duct stones. If the lesions were removed during the previous operation but the bile duct stenosis was not treated, the persistent bile duct stenosis leads to stone recurrence. The disease is aggravated when bile duct stenosis is not treated and the narrow bile duct is used for cholangiojejunostomy. (4) Improper choledochojejunostomy. Conventional choledochojejunostomy has technical deficits, and a certain proportion of patients develop postoperative biliary-enteric anastomosis stenosis or/and hilar bile duct stenosis. This is an important cause of the postoperative recurrence of intrahepatic bile duct stones. To treat this kind of stone recurrence, the original biliary-enteric anastomosis is surgically removed, followed by hilar ductoplasty, intrahepatic bile duct exploration, and stone removal. Then, Roux-en-Y hepaticojejunostomy is performed.

## 6. Conclusion

The pathogenesis of primary intrahepatic bile duct stones is closely related to biliary infection and intrahepatic bile duct cholestasis. Based on the etiology and pathogenic mechanism, the surgical procedures for treatment include bile duct exploration with stone extraction, partial hepatectomy, hilar ductoplasty, and Roux-en-Y hepaticojejunostomy. This method can improve long-term outcomes and reduce the postoperative recurrence of stones.

## Figures and Tables

**Table 1:** Demographic and clinical characteristics.

Parameters	*(N)*	%
*Gender*		
Male	(64)	43.24
Female	(84)	56.76

*Stone distribution prior surgery*		
Limited distribution	(93)	62.84
Diffuse stone	(37)	25.00
Unknown	(18)	12.16

*Prior surgical methods*		
Surgical method I	(29)	19.59
Surgical method II	(33)	22.30
Surgical method III	(58)	39.19
Surgical method IV	(28)	18.92

*Stone distribution before this surgery*		
Limited distribution	(76)	51.35

*Exact locations of stones*		
Left lateral lobe bile duct	(22)	28.95
Left hepatic bile duct	(30)	39.47
Right posterior lobe bile duct	(18)	23.68
Right hepatic bile duct	(6)	7.89
^a^Diffuse stone	(72)	48.65

*Current surgical methods*		
Surgical method A	(79)	53.38
Surgical method B	(69)	46.62

*Imaging diagnosis method*		
Ultrasound, CT, MRCP	(135)	91.22
CT, MRCP	(13)	8.78

*γ-Glutamyltransferase*		
Elevation	(143)	96.62
Normal	(5)	3.38

*Alkaline phosphatase*		
Elevation	(146)	98.65
Normal	(2)	1.35

Surgical methods I: bile duct exploration. Surgical methods II: bile duct exploration and Roux-en-Y cholangiojejunostomy. Surgical methods III: bile duct exploration and partial hepatectomy. Surgical methods IV: exploratory laparotomy, bile duct exploration, partial hepatectomy, and Roux-en-Y cholangiojejunostomy. Surgical methods A: bile duct exploration, extrahepatic bile duct resection, partial hepatectomy, hilar ductoplasty, and Roux-en-Y hepaticojejunostomy. Surgical methods B: bile duct exploration, extrahepatic bile duct resection, hilar ductoplasty, and Roux-en-Y hepaticojejunostomy. MRCP, magnetic resonance cholangiopancreatography; CT, computed tomography. ^a^In 72 patients with diffuse stones, intrahepatic stones were distributed in both the left and right hepatic ducts.

**Table 2:** Methods of reoperation in patients with different stone distributions.

	Surgical methodsA	Surgical methodsB	Total
Limited distribution	39 (51.32%)	37 (48.68%)	76
Diffuse stone	40 (55.56%)	32 (44.44%)	72
Total	79 (53.38%)	69 (46.62%)	148

Surgical methods A: bile duct exploration, extrahepatic bile duct resection, partial hepatectomy, hilar ductoplasty, and Roux-en-Y hepaticojejunostomy. Surgical methods B: Bile duct exploration, extrahepatic bile duct resection, hilar ductoplasty, and Roux-en-Y hepaticojejunostomy.

**Table 3:** Postoperative short-term complications during hospitalization and long-term complications during follow-up.

Postoperative complications	*N*
*Short-term complications*	
Bile leakage	6
Abdominal infection	11
Incision delayed healing	11
Abdominal hemorrhage	4

*Long-term complications*	
Upper abdominal discomfort	15
Bloating	17
Bellyache	5
Cholangitis	18
Bile duct stones	7
Bile duct stricture	2
Cholangiocarcinoma	2

**Table 4:** Long-term therapeutic efficacy (*N* = 124).

Parameters	*N*	%
Excellent	86	69.35
Good	20	16.13
Moderate	11	8.87
Poor	7	5.65

## Data Availability

The datasets generated during and/or analyzed during the current study are available from the corresponding author on reasonable request.

## References

[1] Williams E., Beckingham I., El Sayed G. (2017). Updated guideline on the management of common bile duct stones (CBDS). *Gut*.

[2] Kim H. J., Kim J. S., Joo M. K. (2015). Hepatolithiasis and intrahepatic cholangiocarcinoma: a review. *World Journal of Gastroenterology*.

[3] Pausawasdi A., Watanapa P. (1997). Hepatolithiasis: epidemiology and classification. *Hepato-Gastroenterology*.

[4] Peng J. X., Wang L. Z, Diao J. F. (2018). Major hepatectomy for primary hepatolithiasis: a comparative study of laparoscopic versus open treatment. *Surgical Endoscopy*.

[5] Xie A., Fang C., Huang Y., Fan Y., Pan J., Peng F. (2013). Application of three-dimensional reconstruction and visible simulation technique in reoperation of hepatolithiasis. *Journal of Gastroenterology and Hepatology*.

[6] Olsson G. (2017). Aspects on the role of prophylactic procedures to influence post-ERCP complication rates.

[7] Ran X., Yin B., Ma B. (2017). Four major factors contributing to intrahepatic stones. *Gastroenterology research and practice*.

[8] Tazuma S., Unno M., Igarashi Y. (2017). Evidence-based clinical practice guidelines for cholelithiasis 2016. *Journal of Gastroenterology*.

[9] Li Y., Cai J., Wu A. T., Wang Z. J. (2005). Long-term curative effects of combined hepatocholangioplasty with choledochostomy through an isolated jejunum passage on hepatolithiasis complicated by stricture. *Hepatobiliary and Pancreatic Diseases International*.

[10] Zhu Q. D., Zhou M. T., Zhou Q. Q., Shi H. Q., Zhang Q. Y., Yu Z. P. (2014). Diagnosis and surgical treatment of intrahepatic hepatolithiasis combined with cholangiocarcinoma. *World Journal of Surgery*.

[11] Kim H. J., Kim J. S., Suh S. J. (2015). Cholangiocarcinoma risk as long-term outcome after hepatic resection in the hepatolithiasis patients. *World Journal of Surgery*.

[12] Xia H. T., Xin X. L., Yang T., Liu Y., Liang B., Wang J. (2020). Surgical strategy for recurrent common bile duct stones: a 10-year experience of a single center. *Updates Surg*.

[13] Lillemoe K. D., Melton G. B., Cameron J. L. (2000). Postoperative bile duct strictures: management and outcome in the 1990s. *Annals of Surgery*.

[14] DeOliveira M. L., Winter J. M., Schafer M. (2006). Assessment of complications after pancreatic surgery: a novel grading system applied to 633 patients undergoing pancreaticoduodenectomy. *Annals of Surgery*.

[15] Jeng K. S., Sheen I. S., Yang F. S. (1999). Are expandable metallic stents better than conventional methods for treating difficult intrahepatic biliary strictures with recurrent hepatolithiasis?. *Archives of Surgery*.

[16] Tian J., Li J. W., Chen J. (2013). The safety and feasibility of reoperation for the treatment of hepatolithiasis by laparoscopic approach. *Surgical Endoscopy*.

[17] Pu Q., Zhang C., Huang Z., Zeng Y. (2017). Reoperation for recurrent hepatolithiasis: laparotomy versus laparoscopy. *Surgical Endoscopy*.

[18] Catalano O. A., Sahani D. V., Forcione D. G. (2009). Biliary infections: spectrum of imaging findings and management. *RadioGraphics*.

[19] Li X., Zhu K., Zhang L. (2012). Periampullary diverticulum may Be an important factor for the occurrence and recurrence of bile duct stones. *World Journal of Surgery*.

[20] Uchiyama K., Onishi H., Tani M., Kinoshita H., Ueno M., Yamaue H. (2002). Indication and procedure for treatment of hepatolithiasis. *Archives of Surgery*.

[21] Zhou B., Hu J., Zhong Y. (2017). Surgical treatments for patients with recurrent bile duct stones and Oddis sphincter laxity. *Intractable & rare diseases research*.

[22] Xia H. T., Yang T., Liu Y., Liang B., Wang J., Dong J. H. (2018). Proper bile duct flow, rather than radical excision, is the most critical factor determining treatment outcomes of bile duct cysts. *BMC Gastroenterology*.

[23] Xia H. T. (2020). Standardized surgical management for cystic dilation of the bile ducts based on clinical and pathological studies: a narrative review. *Gastroenterology Research and Practice*.

[24] Xia H. T., Meng X. F., Xin X. L. (2021). Resection of extrahepatic bile ducts with partial hepatectomy for treating intra- and extrahepatic hepatolithiasis. *BMC Surgery*.

[25] Xia H.-T., Liu Y., Yang T., Liang B., Wang J., Dong J.-H. (2017). Better long-term outcomes with hilar ductoplasty and a side-to-side Roux-en-Y hepaticojejunostomy. *Journal of Surgical Research*.

[26] Geng Z.-M., Yao Y.-M., Liu Q.-G., Niu X.-J., Liu X.-G. (2005). Mechanism of benign biliary stricture: a morphological and immunohistochemical study. *World Journal of Gastroenterology*.

